# A Receptor Tyrosine Kinase Inhibitor Sensitivity Prediction Model Identifies AXL Dependency in Leukemia

**DOI:** 10.3390/ijms24043830

**Published:** 2023-02-14

**Authors:** Ahmad Nasimian, Lina Al Ashiri, Mehreen Ahmed, Hongzhi Duan, Xiaoyue Zhang, Lars Rönnstrand, Julhash U. Kazi

**Affiliations:** 1 Division of Translational Cancer Research, Department of Laboratory Medicine, Lund University, 22381 Lund, Sweden; 2 Lund Stem Cell Center, Department of Laboratory Medicine, Lund University, 22184 Lund, Sweden; 3 Department of Hematology, Oncology and Radiation Physics, Skåne University Hospital, 22185 Lund, Sweden

**Keywords:** drug resistance, targeted therapy, TabNet, XGBoost, machine learning, drug sensitivity prediction

## Abstract

Despite incredible progress in cancer treatment, therapy resistance remains the leading limiting factor for long-term survival. During drug treatment, several genes are transcriptionally upregulated to mediate drug tolerance. Using highly variable genes and pharmacogenomic data for acute myeloid leukemia (AML), we developed a drug sensitivity prediction model for the receptor tyrosine kinase inhibitor sorafenib and achieved more than 80% prediction accuracy. Furthermore, by using Shapley additive explanations for determining leading features, we identified AXL as an important feature for drug resistance. Drug-resistant patient samples displayed enrichment of protein kinase C (PKC) signaling, which was also identified in sorafenib-treated FLT3-ITD-dependent AML cell lines by a peptide-based kinase profiling assay. Finally, we show that pharmacological inhibition of tyrosine kinase activity enhances AXL expression, phosphorylation of the PKC-substrate cyclic AMP response element binding (CREB) protein, and displays synergy with AXL and PKC inhibitors. Collectively, our data suggest an involvement of AXL in tyrosine kinase inhibitor resistance and link PKC activation as a possible signaling mediator.

## 1. Introduction

The role of receptor tyrosine kinases (RTKs) in oncogenic transformation is well-documented [1]. Several RTKs are mutated or overexpressed in human cancers and are likely to be involved in constitutive activation of survival and proliferative signaling, and thereby, disease progression [2]. Therefore, inhibition of RTKs has become an attractive approach for the treatment of several cancers [3,4]. However, the development of primary and secondary resistance to RTK-targeted therapies limits its clinical application [5,6].

Several mechanisms explaining the development of therapy resistance have been documented during the past decades [7,8,9]. The most studied mechanisms include the selection of clones during drug treatment due to the pre-existing genetic alterations in a rare population or the acquisition of de novo mutations in cancer cells. During the drug treatment, epigenetic modifications can occur, which in turn contribute to alterations in gene expression of tumor suppressors and oncogenes, leading to the activation of cell survival pathways. Furthermore, dynamic fluctuation of gene expression during drug treatment can help cells escape therapy-induced death resulting in the accumulation of drug-tolerant cells [10,11].

Oncogenic mutations in the type III receptor tyrosine kinase FLT3 are the most commonly identified genetic alterations in acute myeloid leukemia (AML) [12]. Those type of mutations have also been reported in other hematological malignancies including acute lymphoblastic leukemia and myeloproliferative disorders [13,14,15]. Oncogenic mutations in FLT3 lead to constitutive activation of several pro-survival pathways including PI3K/AKT, MAPK pathways, and, with some exceptions, the STAT5 pathway [12]. Besides the identification of FLT3 mutations and their importance in pro-survival signaling, FLT3 inhibitors if used as monotherapy have not been as successful as expected. The first-generation FLT3 inhibitors, such as midostaurin, sorafenib, and sunitinib, were primarily developed to target other kinases and were later found to be useful in inhibiting FLT3 [16]. Such inhibitors display a low degree of specificity and higher toxicity profile by inhibiting a wide range of kinases [17]. The next-generation FLT3 inhibitors, e.g., crenolanib, AC220 (quizartinib), and gilteritinib, are more selective for FLT3 but still inhibit several other RTKs [18]. Several of those inhibitors underwent clinal trials and recently midostaurin and gilteritinib were approved by the FDA, and AC220 was approved for use for AML in Japan (a summary can be found in reference [18]).

The mechanisms by which AML patients develop resistance to the targeted therapies have been explored from different points of view. Patients carrying FLT3-ITD mutations develop resistance to FLT3-targeted therapies predominantly due to secondary mutations in the tyrosine kinase domain and also likely due to the activation of parallel survival signaling pathways during drug treatment [12,18,19,20].

In this study, we developed a machine learning model for drug sensitivity prediction and identified AXL as an important feature. We further show that AXL expression is upregulated during drug treatment and identify a possible link between protein kinase C activity and the regulation of AXL expression.

## 2. Results

### 2.1. Development of a TabNet Model to Predict Sorafenib Sensitivity in Leukemia

**Figure 1:**
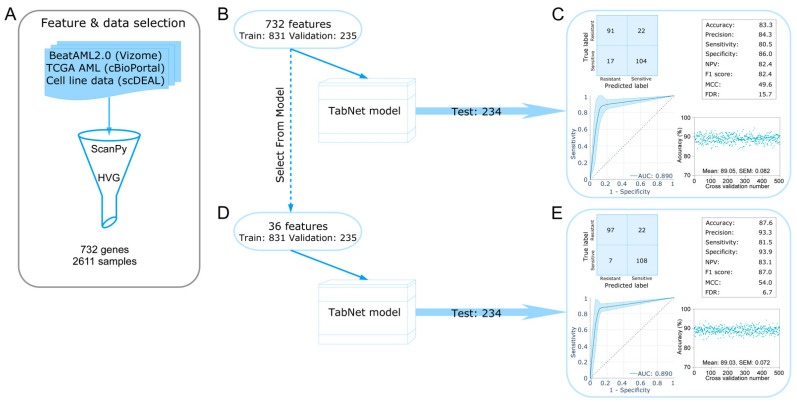
Development of deep learning models for tabular data. (**A**) Features were selected using the “highly variable genes” function from ScanPy. (**B**) Selected features (732) were used to develop a TabNet model. The model was built using 831 training samples and 235 validation samples and then was tested using 234 test samples. (**C**) The models’ performance was checked using a confusion matrix and several quality scores. Furthermore, from all samples (1300), we randomly sampled 20% of test samples 500 different times, and the rest of the 80% samples were used to build TabNet models. The ROC curve and accuracy scores represent results from all 500 tests. (**D**,**E**) A similar test was performed as described in (**B**) and (**C**) using 36 features selected using the “Select From Model” function from Sklearn feature selection.

Several clinical trials have been initiated with the first-generation FLT3 inhibitor sorafenib for maintenance after stem cell transplantation [21], in combination with standard chemotherapy [22], and in combination with other drugs [23]. However, similar to other tyrosine kinase inhibitors, patients in many cases display sorafenib resistance due to the activation of parallel signaling pathways [20,24,25]. The elegant BeatAML studies used sorafenib to treat ex vivo 385 FLT3-ITD negative and 118 FLT3-ITD positive AML samples [26,27]. As shown in Supplementary Figure S1, we observed mixed responses to both FLT3-ITD positive and negative samples from BeatAML studies to sorafenib, although as expected, FLT3-ITD positive samples showed a significantly higher response. Our results suggest that the presence of FLT3-ITD does not exclusively predict sorafenib response. Therefore, we first aimed to develop a tool to predict sorafenib response in AML. We used pharmacogenomic and transcriptomics data from BeatAML, TCGA, and cell line studies. To identify genes that are consistently deregulated in AML we used highly variable genes (using ScanPy [28]) selected from BeatAML and TCGA studies. We then excluded genes that are absent in cell line data (Figure 1A). We identified a list of 732 genes that were then used to develop a binary classification model using a recently developed deep learning algorithm for tabular data (TabNet [29]). We used 1066 samples to train the TabNet model (831 samples for training and 235 samples for validation), and 234 samples for testing the model (Figure 1B). The model predicted 91 samples as sorafenib-resistant (out of 113 samples, 80.5% sensitivity) and 104 samples as sorafenib-sensitive (out of 121 samples, 86.0% specificity) (Figure 1C). Overall, the model achieved 83.3% accuracy and 84.3% precision. We have also tested the model by randomly sampling test samples in 500 different ways and achieved an average area under the curve (AUC) of 0.89 from the receiver operating characteristic (ROC) curve and 89.05% average accuracy. Furthermore, we attempted to reduce the number of features using the “Select From Model” function from the Sklearn feature selection [30], which allowed us to reduce the number of features to 36 (Figure 1D). The model developed using 36 features showed a slight improvement in terms of accuracy (87.6%), precision (93.3), and specificity (93.9) scores. However, when tested with random sampling, performance scores were similar to that of 732 features (Figure 1E).

### 2.2. Shapley Additive Explanations (SHAP) Identify AXL and HTRA4 as Important Features

**Figure 2:**
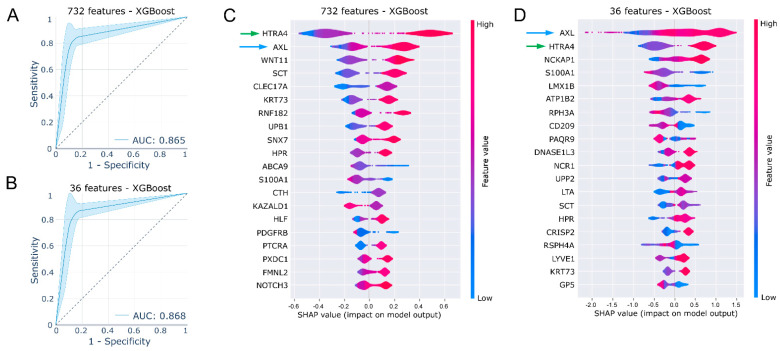
Identification of leading predictors using XGBoost and SHAP. (**A**,**B**) Performance of XGBoost models was checked by random sampling 20% of test samples 500 different times, and the rest of the 80% samples were used to build the models. The ROC curve represents the results from all 500 tests. (**C**,**D**) The SHAP explainer was used to determine SHAP values from test samples. XGBoost was used as the model.

Next, we applied the XGBoost classifier to check the predictive performance of the two sets of features described above. XGBoost is one of the most popular tree-based ensemble algorithms that has been widely used for classifications [31] and can be also used to identify important features when combined with Shapley additive explanations (SHAP) [32]. We used a random sampling method to sample test data (20%) from 1300 labeled samples. XGBoost models were built using 80% data and then tested on the corresponding 20% of the data. Models built using both 732 (Figure 2A) and 36 (Figure 2B) features were equally good as shown in average AUC scores in ROC curves. We then applied SHAP to identify leading predictors. We used both 732 and 36 features and observed that AXL and HTRA4 scored highest in both models (Figure 2C,D). The HTRA4 gene encodes a human protease that belong to the mammalian high temperature requirement A (HtrA) family. HtrA family proteins are involved in several cellular processes, including apoptosis and unfolded protein stress response [33]. A recent study suggested that the expression of HTRA4 enhances the effect of chemotherapy in breast and lung cancer cell lines [34]. AXL, a member of the TAM receptor tyrosine kinase family (TYRO3, AXL, and MER), is well known for its roles in cell proliferation, migration, invasion, and survival. The expression of AXL can also contribute to therapy resistance in many cancer types [35]. While HTRA4 expression may be important for sorafenib sensitivity prediction, AXL is well known for its role in therapy resistance [36]. 

### 2.3. Sorafenib Activates Protein Kinase C Signaling

**Figure 3:**
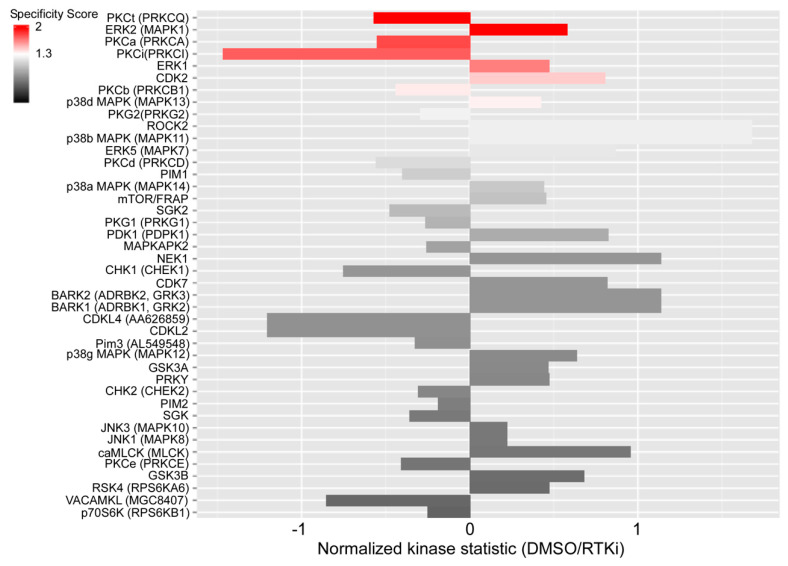
Regulation of serine/threonine signaling during tyrosine kinase inhibition. MOLM-13 cells were treated with DMSO or sorafenib for four hours before lysis. Lysates were applied to the Pamgene serine/threonine kinase profiling assays.

Next, we analyzed the enrichment of genes in sorafenib-resistant patients to map them on pathways in cancer using the BeatAML dataset. A sample was categorized as resistant or sensitive if the sample was both experimentally and predicted (by both TabNet models) in the same category. Using such criteria, 140 samples were labeled as sensitive and 85 samples were labeled as resistant. We mapped deregulated genes on KEGG [37] “Pathways in cancer” using iDEP [38] that uses Pathview [39] for mapping. Supplementary Figure S2 shows enriched pathways in resistant samples. We used a two-fold cut-off (scale shows log_2_ values) and observed enrichment of several serine/threonine kinase pathways. In a recent study, we have shown that pathways that are involved in therapy resistance were deregulated in the initial drug exposure [11]. Therefore, we treated an AML cell line (MOLM-13) with sorafenib for four hours and measured the activation of serine/threonine kinases using a peptide-substrate-based kinase profiling assay. MOLM-13 cells express a copy of FLT3-WT and a copy of FLT3-ITD, and therefore display constitutive activation of FLT3 downstream signaling pathways. We observed that while sorafenib suppressed ERK1/2 activation, which is one of the downstream effectors of FLT3 signaling, the activity of several protein kinase C (PKC) isoforms, including the classical isoform PKCα, the novel isoform PKCθ and the atypical isoform PKCι, were enhanced (Figure 3). PKC family members are involved in various cellular events including cell proliferation, survival, differentiation, apoptosis, and therapy resistance [40,41,42,43]. We observed the enrichment of at least two signaling pathways that activate PKC isoforms in pathway enrichment data (Supplementary Figure S2). Taken together, our data indicate a role of PKC isoforms in sorafenib resistance.

### 2.4. Inhibition of the Receptor Tyrosine Kinase FLT3 Results in Transcriptional Upregulation of AXL

**Figure 4:**
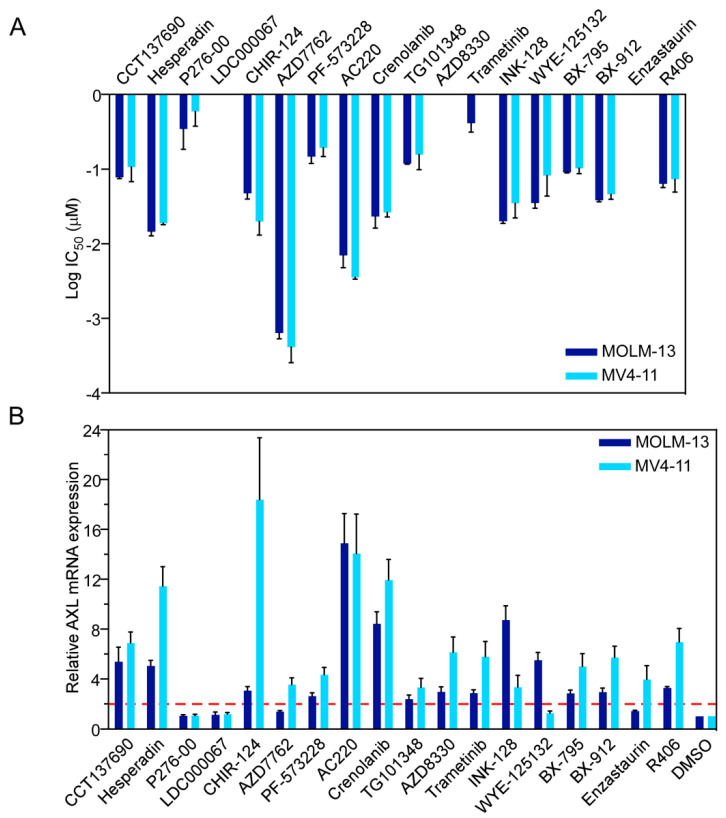
Drug-induced AXL expression in AML cell lines. (**A**) MV4-11 and MOLM-13 cells were treated with different concentrations of kinase inhibitors for 48 h. Cell viability was measured using PrestoBlue. (**B**) MV4-11 and MOLM-13 cells were treated with different kinase inhibitors for 24 h with respective EC_50_ values and AXL expression was measured using RT-qPCR.

Previously, we have demonstrated that dexamethasone-resistant B-cell acute lymphoblastic leukemia cells that express the oncogenic mutant of FLT3 display upregulation of AXL expression upon inhibition of FLT3 [44]. A cross-talk between FLT3-ITD and AXL has also been reported where AXL was identified as a positive regulator of FLT3-ITD [45]. Furthermore, a recent study suggested that AXL expression is upregulated in AC220-resistant cells and cells display sensitivity to AXL-targeted therapy [46]. These studies demonstrate a link between FLT3-ITD activity and AXL expression. To understand how targeted inhibition of FLT3 regulates global transcriptomics in AML cells, we analyzed global gene expression changes using microarray. MOLM-13 and MV4-11 cell lines were treated with AC220 for six hours to capture the early regulations in transcription. The total mRNA from inhibitor-treated cells was analyzed by microarray. As expected, AXL expression was upregulated in inhibitor-treated cells (Supplementary Figure S3A). Besides AXL, 225 genes were upregulated in MV4-11 and 145 genes in MOLM-13 (Supplementary Figure S3B), of which 86 genes were upregulated in both cell lines (Supplementary Figure S3C). Among those 86 genes, several of them are regulators of gene transcriptions (such as ARID5B, BTG2, CREBRF, HBP1, PRDM1, CALCOCO1, CARF, CIITA, HDAC5, MAP2K6, STAT2, TXNIP, TP53INP1, ZNF836, and ZNF846) (Supplementary Figure S3D). As we identified AXL as an important factor contributing to sorafenib response prediction and AXL expression was upregulated during AC220 treatment, we aimed to identify kinases that are involved in the regulation of AXL expression. We used MV4-11 and MOLM-13 cells that have constitutive activation of several pro-survival pathways due to the expression of FLT3-ITD [12,47,48,49,50]. These cells are highly responsive to FLT3-targeting drugs, such as AC220 and crenolanib, but also display sensitivity to a different extent to several pathway-specific inhibitors that target cell cycle regulatory kinases, mitogen-activated protein kinases (MAPKs), the PI3K/mTOR pathway, protein kinase C (PKC), and Janus tyrosine kinase (JAK) (Figure 4A and Supplementary Figure S4A,B). Several of these kinases, such as SYK, PKC, PI3K/mTOR, MAPK, and cell cycle kinases, are direct or indirect effectors of FLT3 signaling [12,50,51,52,53,54,55]. Therefore, inhibition of FLT3 downstream effectors may cooperate in growth inhibition by abrogating FLT3-ITD-mediated survival signaling. However, besides the strong growth inhibition, kinase inhibitors targeting FLT3 downstream effectors were poor in activating increased AXL transcription in both cell lines, as compared to FLT3-targeted inhibition (Figure 4B). The kinase inhibitors that displayed a considerable effect on AXL expression, e.g., hesperadin, may also target FLT3, since several other aurora kinase inhibitors are potential inhibitors of FLT3 [56]. Therefore, it is likely that transcriptional induction of AXL is caused by the direct inhibition of FLT3 and not by its downstream signaling components. However, we cannot exclude the possibility of the involvement of other tyrosine kinases, as inhibitors targeting FLT3 may also inhibit other tyrosine kinases due to similarity in the kinase domain. Furthermore, we observed that AXL expression was dynamically regulated upon FLT3 inhibition (Supplementary Figure S4C). The expression of AXL enhanced over time and reached its highest level after 16 h before declining. Upregulation was also observed at the protein level (Supplementary Figure S4D).

### 2.5. An Inhibitor Targeting FLT3 Displays Synergy with AXL and PKC Inhibitors

**Figure 5:**
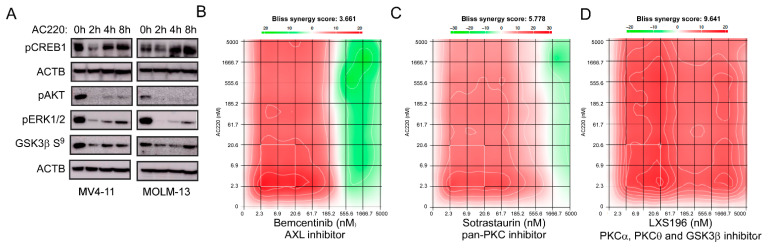
FLT3 inhibition enhances CREB phosphorylation and displays synergy with AXL and PKC inhibitors. (**A**) MV4-11 and MOLM-13 cells were treated with AC220 for different time points before lysis. Lysates were used to separate by SDS-PAGE following western blotting using different antibodies. (**B**–**D**) MV4-11 cells were treated with a single drug or a 1:1 drug combination for 48 h. Relative inhibition was used to calculate the synergy as described in material methods. A positive BLISS score represents the synergy (single drug response curves are presented in the Supplementary Figure S5).

As we observed sorafenib-induced PKC activity in MOLM-13 cells, we checked whether the regulation of PKC activity is a result of the inhibition of FLT3-ITD. We measured the phosphorylation of cyclic AMP response element binding (CREB) protein and observed that CREB phosphorylation was enhanced during the AC220 treatment (Figure 5A). Furthermore, MV4-11 and MOLM13 cells displayed downregulation of AKT, ERK1/2, and GSK3β (Ser 9) phosphorylation in AC220-treated cells (Figure 5A). CREB is a substrate of several serine/threonine kinases including PKC [57,58]. Since we observed that several CREB-activating kinases, such as AKT [59], ERK [60], and GSK3β [61], were inhibited by AC220, it is likely that enhanced CREB phosphorylation was mediated by PKC. A recent report also suggested that AXL expression was regulated by serine/threonine protein kinase PKCα in triple-negative breast cancer [62]. Furthermore, like the AXL inhibitor bemcentinib (Figure 5B), pan-PKC inhibitor sotrastaurin, and LXS196 (inhibits PKCα, PKCθ, and GSK3β) displayed considerable synergy with AC220 (Figure 5C,D), suggesting possible involvement of PKC isoforms in AXL expression.

## 3. Discussion

In this study, we identified two gene signatures that can be used to develop machine-learning models for the prediction of tyrosine kinases inhibitor sorafenib sensitivity. The first gene signature (consisting of 732 genes) was identified using ScanPy’s “highly variable gene” function and the second gene signature (36 genes) was short-listed from the first gene signature by applying the Sklearn feature selection method. We developed a deep tabular data learning model using TabNet and gradient-boosted tree-based models using XGBoost. Both models displayed a similar performance. Furthermore, using Shapley additive explanations we identified AXL as a leading feature.

The TAM family receptor tyrosine kinases are well-studied for their role in leukemia [63]. More specifically, AXL has been shown to be involved in cell proliferation, survival, tumor metastasis, stem cell phenotype, and more importantly, therapy resistance [64]. Virtually all types of cancer treatment, such as chemotherapy, radiotherapy, and targeted therapy, can induce AXL expression, resulting in resistance to treatment. For example, chemotherapy-induced AXL expression was reported in AML [65], lung cancer [66], prostate cancer [67], ovarian cancer [68], and endometrial cancer [69] as conferring therapy resistance. Recently, a series of studies linked AXL to several receptor tyrosine kinase inhibitors including EGFR inhibitors in lung cancer [70], head and neck cancer [71] and colorectal cancer [72], HER2 inhibitors in breast cancer [73], and FLT3 inhibitors in AML [74]. Identification of AXL as a leading feature in the sorafenib sensitivity prediction model further emphasizes its importance in resistance to receptor tyrosine kinase inhibitors.

Several mechanisms by which AXL contributes to the receptor tyrosine kinase inhibitor resistance have been proposed. In AML, cytokine-induced activation of STAT5 enhances AXL expression, leading to AC220 resistance [74]. A natural product, apigenin, reduced AXL expression in ovarian cancer cell lines without affecting IL6 production and STAT3 phosphorylation [75], suggesting the selectivity of the STAT proteins in the regulation of AXL expression. The observation that AML cell lines treated with sorafenib or AC220 enhance PKC activity and AXL expression provides a possible additional mechanism for the regulation of AXL expression. In head and neck and esophageal squamous cell carcinomas, AXL dimerizes with EGFR and activates PKC through PLC*γ* [76], and in breast cancer pharmacological inhibition, PKC resulted in decreased AXL expression [62], suggesting a feedback loop between AXL expression and PKC activation. We observed that pharmacological inhibition of FLT3 in combination with AXL and PKC displays synergy, further suggesting that PKC may be involved in previously reported crosstalk between FLT3 and AXL [45].

In conclusion, we provide a framework of drug sensitivity prediction using a selected gene signature and provide evidence that AXL is involved in resistance to tyrosine kinase inhibitors, linking it to PKC activation as a possible signaling mediator.

## 4. Materials and Methods

Cell cultures: MOLM-13 and MV4-11were obtained from Deutsche Sammlung von Mikroorganismen und Zellkulturen (DSMZ, Braunschweig, Germany). The cell lines were cultured in RMPI-1640. All media were supplemented with 10% heat-inactivated fetal bovine serum (FBS), 100 units/mL penicillin, and 100 µg/mL streptomycin. Cells were maintained at 37 °C with 5% CO_2_ and regularly tested for mycoplasma contamination. All inhibitors were provided by Selleckchem and were dissolved in DMSO according to the manufacturer’s instructions. The final concentration of DMSO in cell cultures never exceeded 0.1%.

Cell viability assay: Cell viability assays were performed using PrestoBlue (Thermo Fisher Scientific, Waltham, MA, USA). Cells (1.8 × 10^4^) were plated into 96-well microplates in 100 μL cell culture medium with increasing doses of inhibitors for 48 h. For analysis, 10 μL PrestoBlue was added to each well, and plates were incubated for 3 h before reading in a microplate reader (excitation 530 nm, emission 590 nm; BioTek Instruments Inc., Winooski, VT, USA). Non-linear regression analysis was used to determine IC_50_.

Real-time quantitative PCR (RT-qPCR): Total RNA was isolated using the RNeasy Mini Kit following the manufacturer’s instructions (Qiagen, Venlo, Netherlands). Total RNA was measured using Nanodrop 2000 Spectrophotometer (Thermo Fisher Scientific). RNA was reverse-transcribed to cDNA using a High-Capacity cDNA Reverse Transcription Kit (Thermo Fisher Scientific) according to the manufacturer’s instructions. Gene expression was assessed using the TaqMan Gene Expression Master Mix and gene-specific probes (Thermo Fisher Scientific). AXL gene expression levels were normalized against the endogenous housekeeping genes GAPDH, β-actin, and α-tubulin. Normalization of gene expression was carried out by ΔΔCt analysis.

Western blot analysis: Cells were lysed in 1% Triton X-100 lysis buffer supplemented with phosphatase/protease inhibitors (Trasylol, PMSF, and Na_3_VO_4_). Protein concentration was determined using bicinchoninic acid (BCA) protein assay (Thermo Fisher Scientific). Lysates were mixed with SDS loading buffer and heated to 96 °C for 5 min. Equal amounts of protein (20 µg) were subjected to SDS-PAGE. Gels were transferred to polyvinylidene difluoride (PVDF) membranes, blocked with 5% non-fat dry milk, and immunoblotted with primary antibodies. The anti-pERK1/2 (1:200), pCREB1 (1:1000), and anti-β-actin (1:5000) antibodies were obtained from Santa Cruz Biotechnology (Dallas, TX, USA). Anti-pAKT Ser473 (1:5000) was obtained from Cell Signaling Technology (Danvers, MA, USA), Anti-pGSK3β (Ser9; 1:1000) was obtained from Thermo Fisher Scientific. Blots were next incubated with a secondary antibody conjugated with horseradish peroxidase (HRP) and developed using a Luminata Forte Western HRP substrate (Millipore, Burlington, MA, USA) and Amersham Imager 600 (GE Healthcare, Chicago, IL, USA).

Machine learning models: A brief description of machine learning models was described previously [77,78]. Pharmacogenomic data were collected from BeatAML, TCGA, and scDEAL studies. To develop binary classification models, Jupyter Notebook was used. The TabNet model was developed on CUDA and all other models were developed on the CPU.

## Data Availability

All raw data are available upon request.

## Supplementary Materials

The following supporting information can be downloaded at: https://www.mdpi.com/article/10.3390/ijms24043830/s1.
