# Supplementary material for: A Receptor Tyrosine Kinase Inhibitor Sensitivity Prediction Model Identifies AXL Dependency in Leukemia
Source: Int J Mol Sci. 2023 Feb 14;24(4):3830. doi: 10.3390/ijms24043830 (PMC9959897; doi:10.3390/ijms24043830)

Supplementary figure S1. Sorafenib data was collected from BeatAML2.0 (vizome.org).

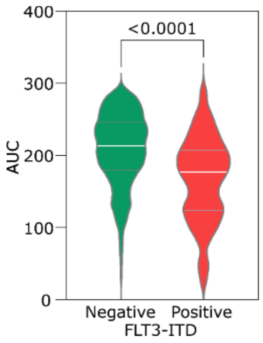

Supplementary figure S2. A pathway map was generated using iDEP.96.

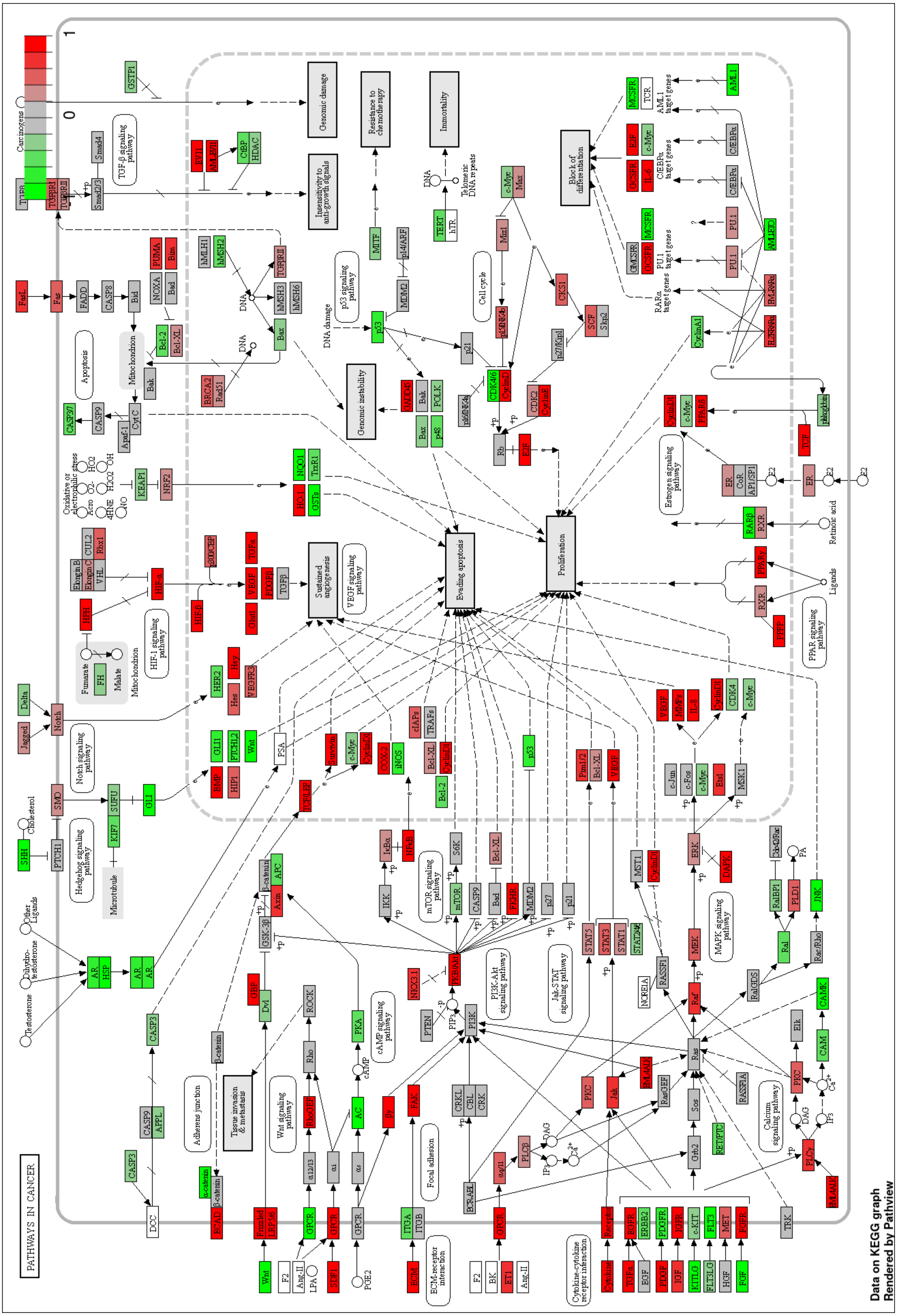

Supplementary figure S3. Global transcriptional changes during drug treatment. MV4-11 and MOLM-13 cells were treated with AC220 for six hours before extraction of total RNA. (A) AXL expression in DMSO vs AC220 treated cells. (B) Upregulated and downregulated genes in DMSO vs AC220 treated cells. (C) Common upregulated genes in MV4-11 and MOLM-13 cells. (D) Heatmap showing upregulated genes in AC220 treated cells compared to DMSO treated cells.

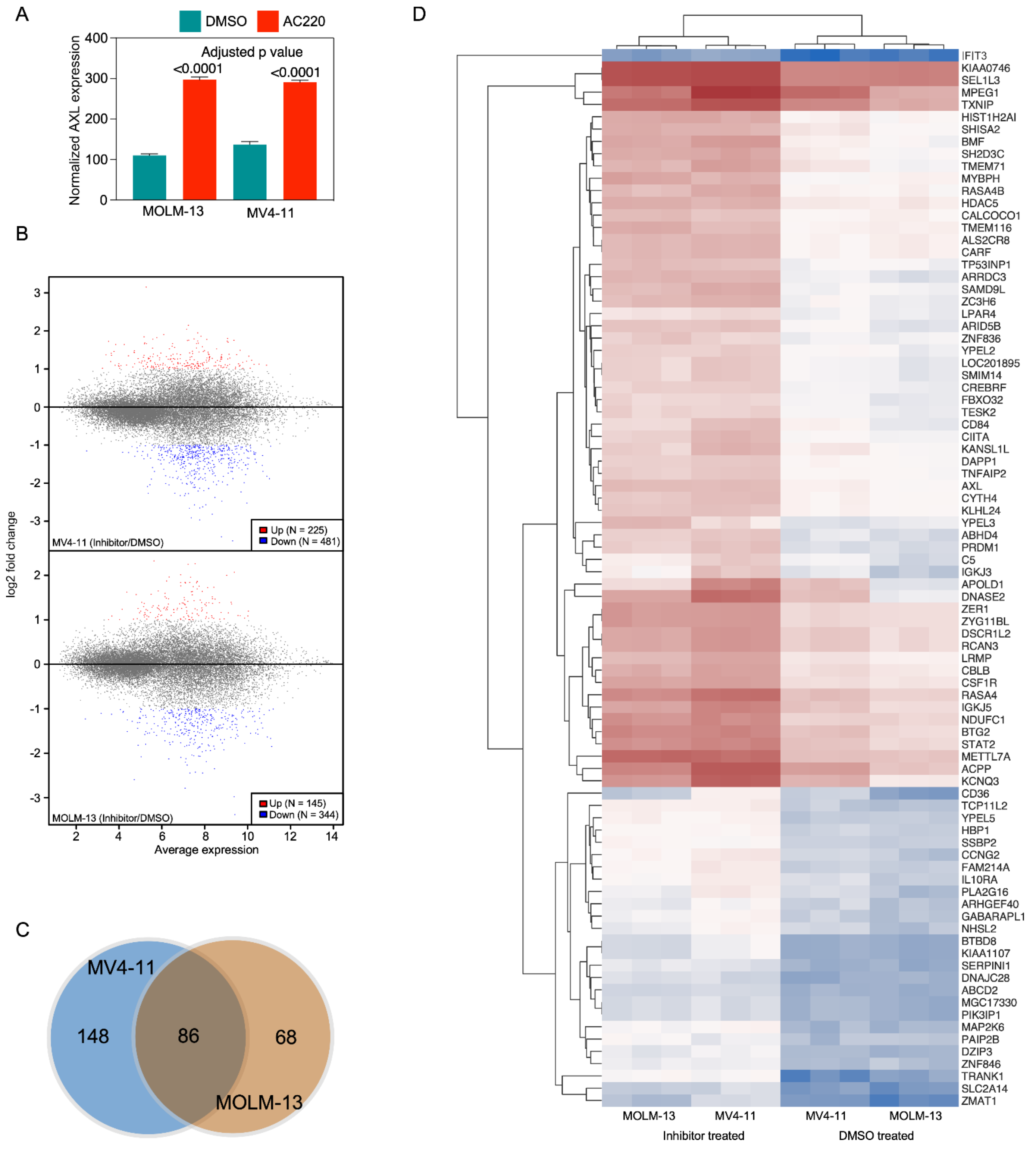

Supplementary figure S4. Transcriptional regulation of AXL during tyrosine kinase inhibition. (A-B) MV4-11 and MOLM-13 cells were treated with different concentrations of kinase inhibitors for 48h. Cell viability was measured using PrestoBlue. (C) Cells were treated with 5 nM AC220 for 0-48h. AXL expression was measured using RT-qPCR. (D) MV4-11 cells were treated with DMSO or specific drugs (using IC_{50} concentration) overnight before lysis. Lysates were used for SDS-PAGE and western blotting.

A

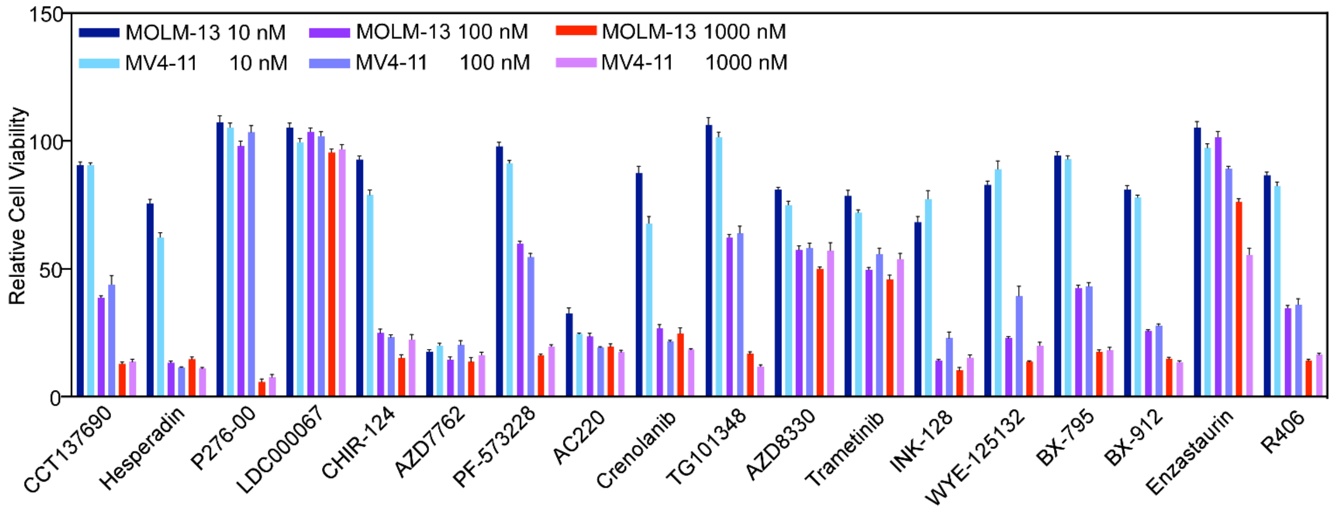

B

Inhibitor	Primary Target	IC_{50} (nM) MOLM-13	MV4-11
CCT137690	Aurora A/B/C	77	114
Hesperadin	Aurora B	15	19
P276-00	CDK1/4/9	392	628
LDC000067	CDK9	>1000	>1000
CHIR-124	CHK1	48	21
AZD7762	CHK1/2	0.65	0.45
PF-573228	FAK	149	198
AC220	FLT3	7.3	3.5
Crenolanib	PDGFR/FLT3	24	26
TG101348	JAK2	118	169
AZD8330	MEK1/2	>1000	>1000
Trametinib	MEK1/2	419	>1000
INK-128	mTOR	20	37
WYE-125132	mTOR	35	92
BX-795	PDK1	91	104
BX-912	PDK1	38	46
Enzastaurin	PKC	>1000	>1000
R406	SYK	64	77

C

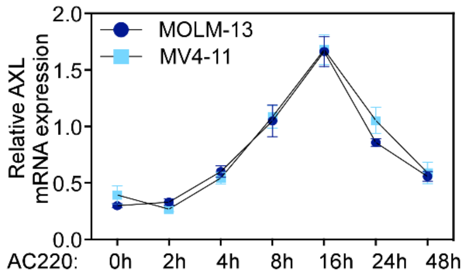

D

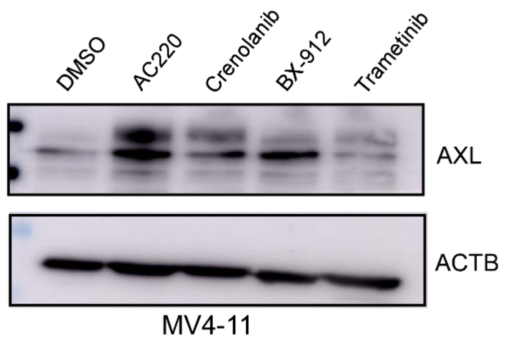

Supplementary figure S5. MV4-11 cells were treated with a single drug for 48h and % of inhibition was plotted using GraphPad.

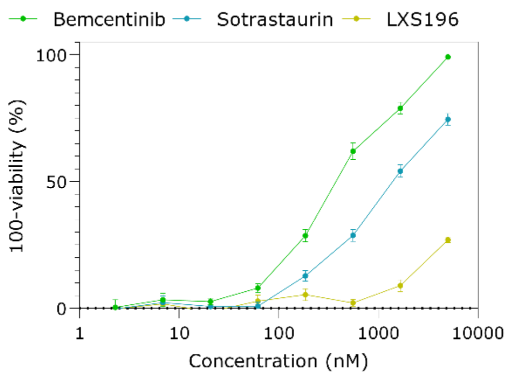

Supplement: Supplementary file 1 [file ijms-24-03830-s001.zip › ijms-2188328-supplementary.pdf]
